# The Role of Maternal Smoking in Effect of Fetal Growth Restriction on Poor Scholastic Achievement in Elementary School

**DOI:** 10.3390/ijerph9020408

**Published:** 2012-01-27

**Authors:** Igor Burstyn, Stefan Kuhle, Alexander C. Allen, Paul Veugelers

**Affiliations:** 1 Department of Environmental and Occupational Health, School of Public Health, Drexel University, 1505 Race Street, Philadelphia, PA 19102, USA; 2 Department of Public Health Sciences, School of Public Health, University of Alberta, Edmonton, Alberta, Canada; Email: skuhle@ualberta.ca (S.K.); paulus.veugelers@ualberta.ca (P.V.); 3 Division of Neonatal-Perinatal Medicine, Perinatal Epidemiology Research Unit, Dalhousie University & IWK Health Centre, Halifax, Nova Scotia, Canada; Email: alexander.allen@iwk.nshealth.ca

**Keywords:** fetal growth retardation, tobacco smoking, maternal exposure, educational achievement, retrospective cohort study, cross-sectional sample

## Abstract

Fetal growth restriction and maternal smoking during pregnancy are independently implicated in lowering intellectual attainment in children. We hypothesized that only reduction of fetal growth that is attributable to extrinsic causes (e.g., maternal smoking) affects intellectual development of a child. Cross-sectional survey of 3,739 students in Nova Scotia (Canada) in 2003 was linked with the perinatal database, parental interviews on socio-demographic factors and the performance on standardized tests when primarily 11–12 years of age, thereby forming a retrospective cohort. Data was analyzed using hierarchical logistic regression with correction for clustering of children within schools. The risk of poor test result among children born small-for-gestational-age (SGA) to mothers who smoked was 29.4%, higher than in any other strata of maternal smoking and fetal growth. The adjusted odds ratio among SGA children born to mothers who smoked was the only one elevated compared to children who were not growth restricted and born to mothers who did not smoke (17.0%, OR = 1.46, 95% CI 1.02, 2.09). Other perinatal, maternal and socio-demographic factors did not alter this pattern of effect modification. Heterogeneity of etiology of fetal growth restriction should be consider in studies that address examine its impact on health over life course.

## 1. Introduction

Fetal growth restriction is a clinical manifestation of diverse aetiologies with multiple consequences to health [1]. A neonate can fail to achieve ‘average’ birth weight appropriate for their sex and gestational age due to variety of causes. The leading intrinsic (and non-modifiable) factor that determines fetal size at birth is the mother’s stature [1]. The most common environmental (and therefore potentially modifiable) cause of fetal growth restriction is maternal smoking. We adopt the term ‘extrinsic’ to refer to causes of fetal growth restriction that are due to external environmental influences, to make a distinction from the normal ‘intrinsic’ fluctuations in physiology that lead to children attaining different size/weight at birth. Whereas the modifiable causes are typically attributed to some pathology (such as fetal hypoxia and malnutrition due to action of nicotine to restrict efficiency of placenta) [2], the intrinsic causes may simply reflect natural variation in a ‘healthy’ population. Maternal smoking can be considered to be as causally related to fetal growth restriction because of consistency among various studies, evidence for the dose-response relationship, effects of smoking cessation in reducing these effects and a valid biological explanation [3].

Restricted fetal growth in general has been associated with lower intellectual attainment in childhood reflected in poor scholastic achievement [4,5,6,7], that can be preceded by cognitive delay in early childhood [8]. These effects appear to be more severe for children born prematurely [9]. However, the mechanisms that may mediate this effect are uncertain and there is paucity of data that can illuminate such question that is nonetheless important for devising an intervention. There is inconsistent evidence from studies of siblings on the extent to which intrinsic variation in fetal growth affect intelligence that may well be attributable to complex interplay of intrinsic and background factors [10,11,12]. The role of prenatal exposure to tobacco smoke *per se* in a child’s intellectual development appears to be still unclear [13], although there is emerging evidence on intellectual deficit in children of mothers who smoke during pregnancy [14,15,16], with some doubt as to whether the association is causal [17,18], although evidence in favour of causation is supported by recent report of dose-response association [19]. There is only one study to date that directly tested whether birth weight mediated the association between cognitive abilities of children and maternal smoking [20]. The authors observed that in a sample of 1,544 3.5 year-old children from Quebec (Canada) there was indeed evidence of mediation by birth weight (inaccurate measure of fetal growth [21]) of the effect of maternal smoking on short-term memory and verbal abilities [20]. However, in the majority of studies the possibility of synergistic effects of maternal smoking and fetal growth on intellectual development is poorly addressed. Most studies focused on isolating the effect of one factor *versus* the other, without considering that they may in fact interact. If there is indeed an effect modification of fetal growth on the intellectual development of children through maternal smoking, then the heterogeneity in the literature on separate effects of maternal smoking and fetal growth on intellectual/cognitive development of children may be explained. However, to be able to address such a research question, a large population-based dataset with a high quality of data on maternal and socio-demographic confounders and robust measures of intellectual development is required. We addressed the research question within a study conducted in the Canadian province of Nova Scotia that linked population-based data on the perinatal phase, socio-demographic factors, and academic performance in elementary school children [22,23].

Specifically, we seek to test the hypothesis that only reduction of fetal growth that is attributable to extrinsic/pathologic causes that induce fetal hypoxia will have a detrimental effect on a child’s intellectual development. Therefore, the research question is whether fetal growth restriction with environmental etiology (such as fetal hypoxia and malnutrition due to maternal smoking) affects intellectual development of school-aged children to a different degree than fetal growth restriction attributable to intrinsic maternal factors or factors of unknown etiology. We address the question of involvement of intrinsic risk factors for SGA in child’s intellectual development by examining risk in SGA children born to non-smokers.

## 2. Methods

For this project, a retrospective cohort was assembled by taking data from a cross-sectional population-based survey in grade 5 students in the Canadian province of Nova Scotia in 2003, Children’s Lifestyle and School Performance Study (CLASS) and linking it with the Nova Scotia Atlee Perinatal Database (NSAPD) on the child’s Nova Scotia Health Insurance number. The scholastic performance of participating children in grade 6 was part of CLASS dataset; it constitutes a study of intellectual attainment of children primarily 11–12 years of age. Parents had to provide additional consents to allow linkages with the Nova Scotia Department of Education test results and the NSAPD. The Research Ethics Board at Dalhousie University approved the overall study, data linkage between the survey and provincial perinatal registry, as well as the current analysis. The Joint Data Access Committee further approved the data linkage and current analysis.

CLASS is a survey of grade 5 students (primarily aged 10–11 years) and their parents conducted in spring of 2003. Its methodology has been described in detail elsewhere [23], what follows is a brief description. The school system in Nova Scotia is dominated by public schools; these were invited to participate in the study. The majority of public schools with Grade 5 classes in Nova Scotia consented to take part in CLASS (96%). However, within each school, the average participation rate was 51.1%. Data used for current analyses was obtained from take-home questionnaires completed by parents plus information of average performance of school on the scholastic aptitude tests.

During the autumn of 2003, when CLASS participants were already in grade 6, their reading and writing aptitudes were evaluated in the Elementary Literacy Assessment, a standardized test administered routinely by the Nova Scotia Department of Education [22]. For the purpose of this study, a student’s performance was dichotomized into pass and fail result on either of the two tests. Test scores were available for 92% of the participants. We did not assess any other metrics of intellectual attunement or scholastic achievement.

Perinatal data for the children enrolled in CLASS was obtained from the Nova Scotia Atlee Perinatal Database (NSAPD) through record linkage that was undertaken by the Reproductive Care Program of Nova Scotia. NSAPD collects data for all births in Nova Scotia hospitals. Data in the NSAPD is abstracted from hospital charts and this process is subject to rigorous quality checks [23,24,25,26]. Record linkage was successful for 80% of subjects in CLASS with the major known reason for failure of linkage being birth outside of Nova Scotia (12%). Information obtained from NSAPD on the mother included smoking during pregnancy (collected upon admission to the birth hospital by self-report; yes/no), pre-pregnancy weight, parity, hypertension (yes/no), age, diabetes and marital status. Child data obtained from the NSAPD comprised sex, birth weight and gestational age. Measure of fetal growth, weight for gestational age and sex, was classified as appropriate (AGA), small (SGA) or large (LGA) for gestational age, respectively, based on Canadian reference values [21]. Records that revealed improbable combination of birth weight and gestational age were excluded from analysis (z-score of birth weight standardized to sex and gestational age ≥5 [21], 2%). We did not collect information on ethnicity given the known sensitivities and possible consequences for participation rates. This, however, is not an important limitation of the study because over 95% of Nova Scotia residents are of Caucasian/European decent, so meaningful comparisons across ethnic groups are not possible in this population and where confounding by ethnicity occurs, it would be minimal.

Data was analyzed using hierarchical logistic regression with poor reading or writing score (does not meet *vs.* meets/exceeds expectations) as binary dependent variable, fixed effects for AGA and LGA, maternal smoking and the interaction of the two, as well as potential confounders related to socio-demographic parental and neighbourhood characteristics treated also treated as fixed effects, with correction for clustering of children within schools by introduction of a random school effect. Interaction term in logistic regression is a very specific form that does not necessarily capture effect modification that is more broadly understood as heterogeneity of effects [27]. Therefore, in testing for effect modification of the impact of SGA on scholastic achievement by maternal smoking, we also conducted analyses using variables that are a combination of fetal growth and maternal smoking with non-smoking mother and AGA as reference [28], these models were estimated with and without adjustment for potential confounders, but only fully adjusted models are presented in detail. If there is the hypothesized effect modification, SGA and maternal smoking would be the only effect that confers risk that is different from reference. All analyses were implemented in STATA 11 (Stata Corp, College Station, TX, USA).

## 3. Results

Table 1 suggest that there is a degree of mutual confounding of effects of SGA and maternal smoking on scholastic performance. Being SGA conferred increased risk of at least one poor test score (26.2%) compared to AGA children (19.3%) with odds ratio (OR) 1.46 (95% confidence interval (CI) 1.14, 1.87) in unadjusted analysis. Likewise, maternal smoking conferred increased risk of poor test scores in unadjusted analysis with OR 1.72 (95% CI 1.44, 2.06) relative to children born to non-smokers. However, after correction for all other potential confounders, maternal-smoking-adjusted effect of SGA was attenuated towards the null (OR = 1.20, 95% CI 0.91, 1.57) while though also attenuated, the marginal effect of maternal smoking adjusted for SGA persisted (OR = 1.21, 95% CI 1.00, 1.48). Other notable results include higher risk of poor test result for offspring of younger mothers, those who experienced hypertension, and those with increased parity (Table 1). The association of maternal smoking with elevated risk of SGA was noted (Appendix).

**Table 1:** Relationship of perinatal and maternal factors with poor performance of scholastic aptitude tests (N = 3,739).

	Students	Poor test results		OR1 (95% CI)	OR2 (95% CI)
	Tested				
	N	n	%		
Sex: male	1,765	438	24.8	1	1
Female	1,974	302	15.3	0.53 (0.44, 0.63)	0.46 (0.39, 0.56)
Appropriate for gestational age (AGA)	2,896	560	19.3	1	1
Small for gestational age (SGA)	412	108	26.2	1.46 (1.14, 1.87)	1.20 (0.91, 1.57)
Large for gestational age (LGA)	431	72	16.7	0.86 (0.65, 1.13)	0.92 (0.69, 1.24)
Preterm	185	46	24.9	1.46 (1.02, 2.09)	1.38 (0.94, 2.02)
Breast-feeding					
<1 week	1,476	364	24.7	1	1
1 week–3 months	565	107	18.9	0.70 (0.55, 0.90)	0.83 (0.64, 1.08)
3–6 months	592	82	13.9	0.52 (0.40, 0.68)	0.81 (0.60, 1.08)
>6 months	806	108	13.4	0.48 (0.38, 0.62)	0.73 (0.56, 0.95)
Missing	300	79	26.3	1.22 (0.89, 1.66)	2.92 (1.64,5.20)
*Mother’s characteristics*					
Smoker during pregnancy	1,015	272	26.8	1.72 (1.44, 2.06)	1.21 (1.00, 1.48)
Age (years)					
<20	188	59	31.4	1.95 (1.37, 2.77)	1.75 (1.19, 2.59)
20–24	701	177	25.3	1.45 (1.16, 1.82)	1.31 (1.03, 1.68)
25–29	1,428	265	18.6	1	1
30–34	1,075	184	17.1	0.92 (0.74, 1.15)	1.01 (0.80, 1.27)
35+	347	55	15.9	0.86 (0.62, 1.19)	0.91 (0.64, 1.30)
Pre-pregnancy weight (kg)					
≤60	1,561	300	19.2	1	1
60–70	965	167	17.3	0.89 (0.71, 1.10)	0.96 (0.77, 1.21)
70–80	469	103	22	1.17 (0.90, 1.53)	1.20 (0.90, 1.59)
≥80	382	97	25.4	1.36 (1.03, 1.79)	1.20 (0.89, 1.61)
Missing	362	73	20.2	1.03 (0.76, 1.38)	0.90 (0.66, 1.23)
Hypertension	343	87	25.4	1.45 (1.10, 1.90)	1.44 (1.07, 1.93)
Parity					
1	1,634	300	18.4	1	1
2	1,376	291	21.2	1.21 (1.01, 1.46)	1.43 (1.16, 1.77)
3+	729	149	20.4	1.17 (0.93, 1.47)	1.44 (1.10, 1.88)

OR1: one fixed effect per model with random school effect (280 groups). OR2: as OR1 but with all main fixed effects from Table 1 and Table 2 considered simultaneously.

Examination of socio-demographic characteristics shown (Table 2) that were used to adjust our main associations of interest (Table 1) revealed noteworthy patterns. These suggest that less educated lower income parents have children who do poorly on the tests. It also shows a very strong effect of school-wide failure rate on individual student’s performance. We note that it is legitimate to consider the school’s academic performance as a potential confounder in our analyses as it is likely related to the outcome (chance of obtaining individual poor test score) and exposure of interest though clustering of health-related behaviours such a maternal smoking in catchment areas of particular schools. The patterns of results in adjusted and unadjusted models for the school's academic performance are similar.

**Table 2:** Relationship of socio-demographic factors with poor performance of scholastic aptitude tests (N = 3,739).

	Students	Poor test results		OR1 (95% CI)	OR2 (95% CI)
	Tested				
	N	n	%		
Household Income					
$0–$20,000	291	96	33	3.23 (2.36, 4.43)	1.69 (1.13, 2.51)
$20,001–$40,000	654	166	25.4	2.21 (1.71, 2.86)	1.38 (1.03, 1.84)
$40,001–$60,000	791	132	16.7	1.30 (1.00, 1.69)	0.93 (0.70, 1.23)
> $60,000	1,178	151	12.8	1	1
‘prefer not to answer’	825	195	23.6	1.99 (1.56, 2.55)	1.33 (0.99, 1.79)
Parental education					
Secondary school or less	1,013	279	27.5	2.87 (2.25, 3.65)	1.69 (1.28, 2.24)
College	1,346	278	20.7	2.01 (1.59, 2.55)	1.42 (1.09, 1.84)
University	1,123	125	11.1	1	1
Missing	257	58	22.6	2.34 (1.61, 3.40)	0.69 (0.26, 1.84)
Parents married/common-law	2,899	538	18.6	0.70 (0.56, 0.88)	1.00 (0.76, 1.31)
Missing	275	62	22.6	0.93 (0.64, 1.34)	0.67 (0.28, 1.63)
Neighborhood dwelling value					
Low tertile	1,260	306	24.3	1	1
Medium tertile	1,217	240	19.7	0.75 (0.60, 0.94)	1.12 (0.90, 1.39)
High tertile	1,262	194	15.4	0.58 (0.46, 0.75)	1.24 (0.98, 1.57)
School’s academic performance (% failure)					
<10	912	41	4.5	1	1
10–19	1,318	211	16	4.05 (2.86, 5.72)	3.64 (2.55, 5.19)
20–29	927	238	25.7	7.34 (5.19, 10.4)	6.85 (4.80, 9.78)
30–39	582	250	43	16.0 (11.2, 22.8)	14.1 (9.7, 20.5)

OR1: one fixed effect per model with random school effect (280 groups). OR2: as OR1 but with all main fixed effects from Table 1 and Table 2 considered simultaneously.

Examination of multiplicative interaction of measures of fetal growth and maternal smoking yielded the odds ratio for the joint effect of maternal smoking and SGA on poor test result of 1.40 with narrow 95% CI that excluded null (1.00, 1.96). This effect modification was confirmed in analysis presented in Table 3. It indicates that the risk of poor test result among 211 children who where SGA and born to mothers who smoked was 29.4%, a rate higher than in any other category and almost twice as high as that among AGA children born to non-smoking mothers (17%). The only elevated adjusted odds ratio was among SGA children born to mothers who smoked (OR = 1.46, 95% CI 1.02, 2.09). Although the results suggest that maternal smoking contributed to risk of poor test results across measures of fetal growth, it was clearly not distinguishable from reference category, e.g., for AGA children born to mothers who smoked OR = 1.17, 95% CI 0.94, 1.47. It should also be noted that, although not hypothesised, there was an elevated (the second highest) rate of poor test performance in a small group of 52 LGA children born to mothers who reported to have smoked during pregnancy (28.8%). The lowest rate of test failure was also among children born LGA but to non-smoking mothers (15.0%).

**Table 3:** Effect modification: maternal smoking and fetal growth in association with poor performance of scholastic aptitude tests (N = 3,739).

Combination of exposures			Poor test		OR ^2^	95% CI
Fetal growth ^1^	Maternal smoking	N	n	%		
AGA	No	2,144	365	17.0	reference	
	Yes	752	195	25.9	1.17	0.94, 1.47
SGA	No	201	46	22.9	1.16	0.79, 1.72
	Yes	211	62	29.4	1.46	1.02, 2.09
LGA	No	379	57	15.0	0.86	0.62, 1.20
	Yes	52	15	28.8	1.45	0.75, 2.81

^1^ appropriate for gestational age (AGA), small for gestational age (SGA), large for gestational age (LGA); ^2^ adjusted odds ratios and 95% confidence intervals (CI) as in Table 1 and Table 2.

Our contention that the majority of SGA births among mothers who reported to have smoked is indeed due to maternal smoking appears to be justified. According to Table 3, the risk of SGA birth among smokers is 21.9% and among non-smokers—8.6%. Therefore, we estimate that 61% of all SGA cases are indeed attributable to reported maternal smoking (= [relative risk − 1]/relative risk). 

## 4. Discussion

Observed excess of poor test scores in children 11–12 years born both SGA and to smokers supports our *a prior* hypothesis that poor scholastic achievement is caused by exogenous exposures that produce growth restriction, although clearly we cannot claim that every child in the SGA-smoking category was growth-restricted due to maternal smoking. This is in agreement with a smaller study by Huijbregts *et al*. in another Province of Canada (Quebec) [20] that tested much younger children and reported mediation of the effect of birth weight by maternal smoking on early cognitive ability. Our results were not affected by exclusion of children born preterm (details not shown) and were adjusted for exhaustive list of individual- and neighbourhood-level confounders. Unlike the study of Huijbregts *et al.* [20] we examined effect of fetal growth, rather than just birth weight that is a mixture of growth-restricted and appropriate-for-gestational-age births. The synthesis of these two findings is that it is perhaps not meaningful to ask whether fetal growth, a heterogeneous condition, causes deficit in cognitive abilities and intellectual attainment, but more attention should be paid to consequences of fetal growth restriction of specific aetiologies. It remains our conjecture that extrinsic causes of fetal growth restriction (maternal smoking, nutrition *etc*.) will have more pathological consequences than intrinsic causes such as maternal stature. The question as to whether studied specific scholastic aptitude tests predict later life achievement is relevant to gauging societal impact of studied risk factors and it is unfortunate we do not have any means to address it. We also do not know whether failure on scholastic aptitude tests indicates delay in development at the age of testing or a more fundamental harm that precludes attainment of certain competencies altogether.

Biological interpretation of our result is that *in utero* exposure maternal smoking causes fetal hypoxia and malnutrition [3] which are in turn associated with poor neurodevelopmental outcomes [29,30], with subtle sub-clinical manifestations of these in reduced academic performance. In this sense, fetal growth restriction following maternal smoking during pregnancy may be a marker of sufficiently high exposure to causative agent that leads to events resulting in neurodevelopmental deficit through a separate pathway. This would certainly be consistent with the observation from our results (no excess risk in SGA-non-smoker category) and that of others [10,11,12] that size at birth *per se* is not an indicator of future scholastic achievement/intellectual ability. However, we do not know the extent to which SGA is on the causal pathway between maternal smoking and cognitive function at 11–12 years of age and the matter cannot be settled until heterogeneity of fetal growth restriction is directly addressed in such research.

If our results are not artifact of measurement error and latent confounding, they have clear implication for public health. Although few additional arguments are needed to support reduction or elimination of smoking during pregnancy, perhaps there is room for additional advice to pediatricians and educators to pay particular attention to cognitive development of children who were born SGA to mothers who smoked. These children may require additional interventions to assist them attaining their intellectual and scholastic potential. It is notable that even if mechanism of action that we identified is not correct, this group of children appears to be at elevated risk of failing in school compared to their peers, even after control for neighborhood and individual socio-demographic factors. General intervention to assist these children may be beneficial even if our mechanistic hypothesis is not correct. By focusing such an intervention on subset of children who were born SGA, only approximately half of all children born SGA would be eligible, thereby perhaps reducing the cost of the overall effort. 

The main limitation of our analysis arises from the implicit claim that a SGA child born to a mother who smoked was growth-restricted due to maternal smoking. It is certainly likely that there were a proportion of SGA children born to smokers who would have been born growth-restricted regardless of whether their mothers smoked. Our only assumption is that there were proportionally more SGA children with extrinsic cause of SGA born to smokers than non-smokers. If such misclassification of extrinsic *versus* intrinsic fetal growth restriction was non-differential, we can expect that test of our hypothesis would be biased towards the null [31]. However differential nature of error is difficult to assure since both maternal smoking and optimality of fetal growth are measured with some uncertainty [31]. It must be noted that in calculating attributable fraction in support of the claim that SGA among smokers was indeed attributable to smoking of mothers, we used odds ratio as if it was a relative risk. Given that odds ratios and relative risks are not equivalent for outcomes that are not rare (as is the case for poor test results in our paper), our estimate of attributable fraction of 61% may be inflated. However, as we note below, under-reporting of maternal smoking can have a substantial effect on attributable fraction in the opposite direction. A lesser limitation of our analysis is that maternal smoking was obtained by self-report and recorded merely as a present of abscent [32]. The resulting misclassification of maternal smoking status may have certainly biased our results, with direction of the effect difficult to predict without carrying out formal sensitivity analysis, given that we cannot be sure that exposure misclassification is non-differential [31]. It is likely that due to social desirability bias maternal smoking was under-reported [32] which would tend on average to dilute any true associations that we were able to observe [33,34] and would act to produce an under-estimate of attributable fractions by also under-estimating prevalence of maternal smoking [34]. We are not aware of any assessment of reliability or validity of maternal smoking data in Nova Scotia Atlee Perinatal Database, precluding more detailed analysis of the issue. However, we do note that others have reported lower numbers for (with different methodology) for Nova Scotia in 2005–6: 13.8% [35]. Given declining rates of smoking in Nova Scotia (except for mothers <20 years old) [36], our estimate of maternal smoking in Nova Scotia in 1991–92 is not unrealistic. Another estimate of maternal smoking in Nova Scotia in 1988–92 is consistent with our data and indicates an overall smoking rate of 32.4%, based on examination of records obtained both prenatally and at the time of admission to hospital for delivery [37].

It is also possible that confounding by quality of perinatal care may have affected our results. Women from lower socio-economic status might be more likely to smoke and less likely to receive early and frequent perinatal care. It is also reasonable to suspect and is consistent with results in Table 2 that socio-economic status of parents is related to scholastic achievement. Altogether, this may result in residual confounding since SGA may be due to poorer perinatal care and, under the proposed mechanism, would be more common among smokers. It must be noted that potential for confounding by socio-economic status is reduced in our work be means of collecting detailed information on socio-economic status of studied families. It must be noted that Canada has a public health care system with equal opportunity to health services and most residents of Nova Scotia take advantage of perinatal care programs, hence this form of bias is considered minor in principle.

Selection of subjects into our cross-sectional sample may have biased our findings. The sample does not include children who did not make it to grade 5 or who skipped a grade to be in a grade higher than grade 5 in 2003. Therefore, it is possible that children born to smokers who are also SGA were preferentially excluded from the study if they are indeed at elevated risk of neurodevelopmental deficiency and learning disabilities/deficits. The net results would be to make the observed association of maternal-smoking-SGA with poor scholastic achievement an under-estimate of true effect of this exposure on the risk. As noted in methods, the participants were drawn from all eligible schools, most of which participated; among participating students, record linkage was virtually complete. We do not have detailed data on non-participants because they, by definition, did not consent to record linkage. The only substantial loss of participants was within schools but is unlikely to be related to variables of interest to this analysis. However, this non-response within schools may affect our certainty about extrapolating findings to all students in Nova Scotia. 

The observation that AGA children born to mothers who smoked did not suffer from elevated rates of poor scholastic performance can be explained by supposing that mothers of these children did not smoke enough to cause SGA, but this is a speculation that we cannot substantiate with current data. It was noted in a sample of 1,951 ‘high-risk’ families in the U.S. that maternal smoking of more than a pack of cigarettes/day during pregnancy but not less than that affected behavioral problems of three-year olds beyond influence of confounders [19]. This suggests that maternal smoking during pregnancy has to sufficiently intense to affect behavior in children beyond competing risk factors. Consequently, reducing (not just eliminating) maternal smoking appears to have value in prevention of behavioral problems in children. The result of Boutwell *et al.* [19] also match one interpretation of our findings that sufficiently intense *in utero* exposure to tobacco smoking (*i.e.*, that causing fetal growth restriction) has to occur to alter higher mental functioning of the child. Unfortunately, Boutwell *et al.* [19] did not consider fetal growth and other pregnancy-related factors although among the strength of their approach is control for socio-economic factors though propensity score matching and a sizable population-based sample.

In summary, our results contribute to understanding of how maternal smoking during pregnancy, fetal growth restriction and scholastic achievement may be interrelated. Heterogeneity of etiology of fetal growth restriction should be consider in studies that address examine its health impact. We found support for the notion that extrinsic/environmental *in utero* insult sufficient to cause fetal growth restriction, rather than intrinsic variation in fetal growth, may have lasting consequences for child’s intellectual attainment over the life course.

## Appendix

**Appendix:** Association of maternal smoking with fetal growth (N = 3,739).

Fetal growth	Did not report smoking	Reported smoking	Prevalence in smokers (%)
AGA	2,144	752	26
SGA	201	211	51
LGA	379	52	12
